# CCDC28A deficiency causes head-tail coupling defects and immotility in murine spermatozoa

**DOI:** 10.1038/s41598-024-78453-9

**Published:** 2024-11-05

**Authors:** Nena Stojanovic, Rosario Ortiz Hernández, Nayeli Torres Ramírez, Olga Margarita Echeverría Martínez, Abrahan Hernández Hernández, Hiroki Shibuya

**Affiliations:** 1 Department of Chemistry and Molecular Biology, University of Gothenburg, Gothenburg, 41390 Sweden; 2 Laboratorio de Microscopía Electronica Gerardo Hebert Vázquez Nin, Depto de Biología Celular, Facultad de Ciencias, Universidad Nacional Autónoma de México, México City, México; 3 Laboratorio de Investigación en Patología Experimental, Hospital Infantil de México Federico Gómez, Ciudad de México, 06720 México; 4 Science for Life Laboratory, Department of Cell and Molecular Biology, National Genomics Infrastructure, Karolinska Institute, Stockholm, Sweden; 5 Laboratory for Gametogenesis, RIKEN Center for Biosystems Dynamics Research (BDR), Kobe, Japan; 6 Graduate School of Science, Osaka University, 1-1 Machikaneyama, Toyonaka, 560-0043 Osaka Japan

**Keywords:** CCDC28A, CCDC28B, Flagellum, Spermiogenesis, Sperm, Head-tail coupling apparatus (HTCA), Cilia, Ciliogenesis, Spermatogenesis

## Abstract

Male infertility presents a substantial challenge in reproductive medicine, often attributed to impaired sperm motility. The present study investigates the role of CCDC28A, a protein expressed specifically in male germ cells, whose paralog CCDC28B has been implicated in ciliogenesis. We identify unique expression patterns for CCDC28A and CCDC28B within the mouse testes, where CCDC28A is expressed in germ cells, whereas CCDC28B is expressed in supporting somatic cells. Through knockout mouse models and histological analyses, we reveal that CCDC28A deficiency results in diminished sperm motility and structural aberrations in sperm tails, notably affecting the head-tail coupling apparatus (HTCA), thereby causing male infertility. Fine structural analyses by transmission electron microscopy reveal disruptions at the capitulum-basal plate junction of the HTCA in the CCDC28A mutants. This results in the bending of the head within the neck region, often accompanied by thickening of the tail midpiece. Our discovery demonstrates that CCDC28A plays an essential role in male fertility and sperm tail morphogenesis through the formation of HTCA.

**Supplementary Information:**

The online version contains supplementary material available at 10.1038/s41598-024-78453-9.

## Introduction

Male infertility poses a substantial challenge in reproductive medicine and affects millions of couples worldwide^1^. Impaired sperm motility, often attributed to abnormalities in sperm tail formation and function, is a major cause of male infertility^2^. Understanding the intricate processes underlying spermiogenesis, particularly the development and organisation of the sperm tail, which is essential for sperm movement, is imperative for addressing male infertility caused by impaired sperm motility.

Spermiogenesis, the final phase of spermatogenesis, encompasses a series of transformative events in which haploid round spermatids undergo differentiation involving nuclear condensation, acrosome formation, manchette formation and assembly of the sperm tail (flagellum). The core of the flagellum, called the axoneme, is made up of a structure with “9 + 2” microtubules. The axoneme is surrounded by additional structures, including outer dense fibers (ODFs), a fibrous sheath, and a mitochondrial sheath^3,4^. The coordinated assembly of these structures ensures integrity and functionality of the sperm tail, which is essential for motility and successful fertilisation.

**Fig. 1:**
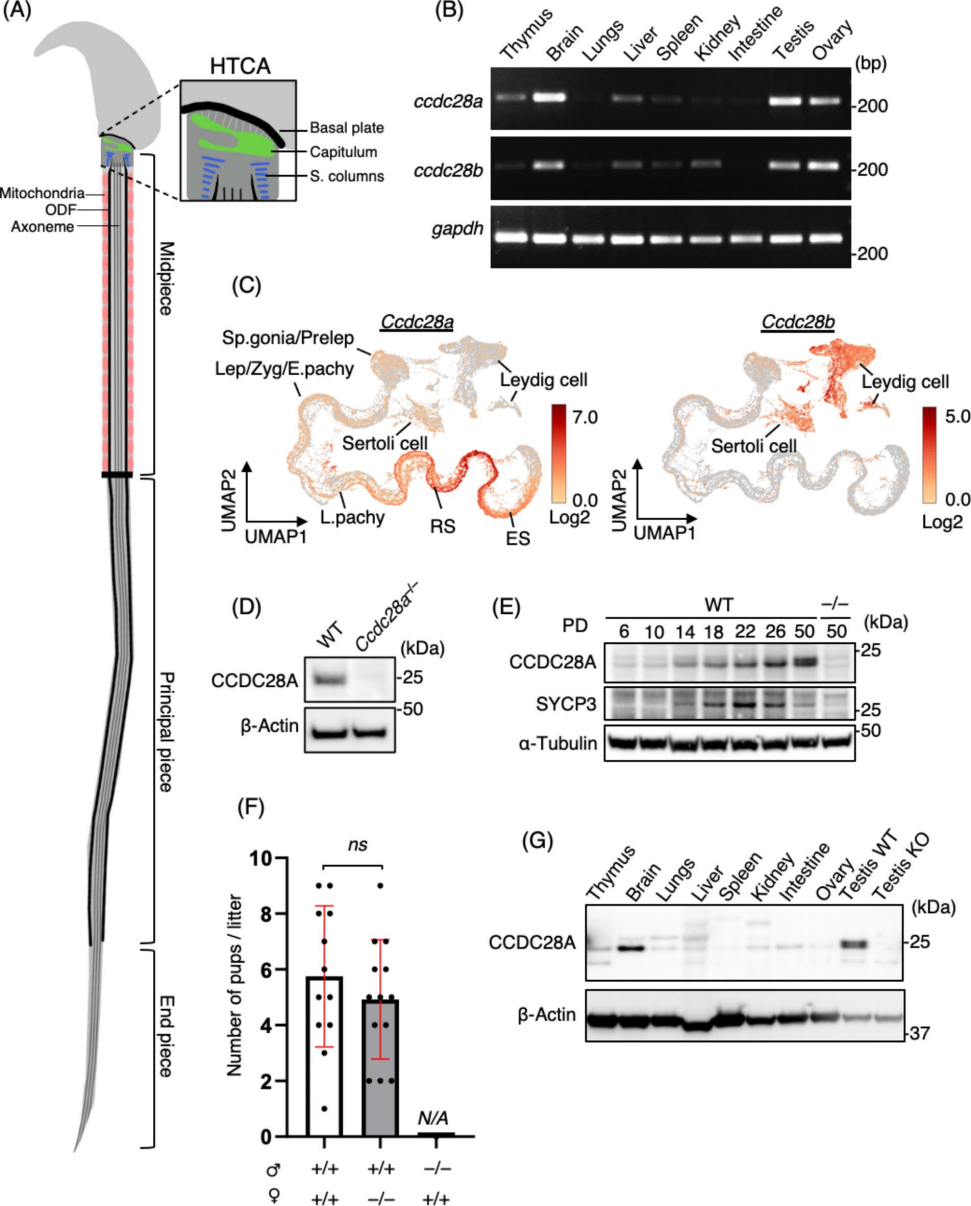
CCDC28A is indispensable for male fertility. (**A**) Schematics displaying the HTCA and organization of the sperm tail. (**B**) Tissue-specific mRNA expression levels of *Ccdc28a* and *Ccdc28b* as assessed by RT-PCR. *Gapdh* was used as a loading control. (**C**) Uniform manifold approximation and projection (UMAP) representation of *Ccdc28a* and *Ccdc28b* expression in mouse testes. Multiplexed single-cell RNA sequencing data from WT mouse testis were used. Spermatogonia, Sp.gonia; preleptotene, Prelep; leptotene, Lep; zygotene, Zyg; early-pachytene, E.Pachy; late-pachytene, L.Pachy; round spermatid, RS; elongated spermatid, ES. (**D**) Western blotting of mouse testis extracts from WT and *Ccdc28a*^*−/−*^ mice. β-actin was blotted as a loading control. Two blots were performed by stripping the same membrane. (**E**) CCDC28A expression in different developmental stages of spermatogenesis assessed by western blotting. α-tubulin was used as a loading control, and SYCP3 as a marker for spermatocytes. The single membrane was cut and blotted with the indicated antibodies. For, CCDC28A, and SYCP3, the same cut membrane was stripped and blotted sequentially. (**F**) Average litter size. Three pairs of PD60 male (♂) and female (♀) mice with the indicated genotypes were mated for six months of continuous breeding. Two-tailed *t*-test was used for statistical analysis. Ns, not significant. N.A. indicates that the data is not applicable for that group owing to the complete sterility. The mean values with standard deviation (SD) are shown. (**G**) Tissue-specific expression of CCDC28A and β-actin analysed by western blotting. *Ccdc28a*^*−/−*^ (KO) testis was used as the negative control. The single membrane was cut and blotted with the indicated antibodies. Source numerical data and unprocessed blots are available in Source data.

The head-tail coupling apparatus (HTCA) serves as a central structure in sperm tail formation, linking the sperm head to the flagellum^5^ (Fig. 1A). The HTCA is essential for maintaining the integrity and functionality of sperm cells during movement and comprises proximal and distal centrioles, associated dense materials and critical structural proteins. Dense materials, such as the capitulum and segmented columns, encase the centrioles. The capitulum links the connecting piece to the sperm head by associating with the implantation fossa at the nuclear surface, and the segmented columns provide structural support to the HTCA by anchoring it to ODFs at their caudal ends^6^. Integral to HTCA formation and function are various structural proteins, including ODF proteins (e.g., ODF1)^7–9^, and proteins specifically localized to the HTCA (e.g., SUN5, PMFBP1, SPATA6, CENTLEIN, CCDC159)^10–15^, which contribute to head-tail coupling and sperm motility. Microtubules and actin filaments form intricate networks within the HTCA, facilitating cargo protein transportation and structural organisation. The microtubule-based intramanchette and intraflagellar transport systems are crucial for delivering proteins and structural components to the developing HTCA, ensuring its proper assembly and functionality^16–19^. Defects in HTCA formation typically result in the separation of the sperm head from the tail during movement, decreased sperm motility and ultimately male infertility^14,15,20–24^.

Coiled-coil domain-containing protein 28 B (CCDC28B) is an evolutionarily conserved protein that functions in ciliogenesis in somatic cells. In humans,* CCDC28B* mutations are associated with Bardet-Biedl syndrome (BBS), a pleiotropic genetic disease caused by impaired primary cilia functions^25,26^. Depletion of CCDC28B in both mammalian cell lines and zebrafish results in impaired ciliogenesis, leading to phenotypic abnormalities akin to ciliopathies, such as BBS^26,27^.

In this study, we explored the role of coiled-coil domain containing protein 28 A (CCDC28A), an evolutionarily conserved paralogue of CCDC28B, in knockout (KO) mouse models. We found that CCDC28A is indispensable for male fertility, although it has been reported that *Ccdc28b *KO mice are fertile^28^. Both CCDC28A and CCDC28B exhibited high mRNA expression levels in the testes; however, only CCDC28A was primarily expressed in male germ cells. We further revealed that CCDC28A is essential for HTCA stability, thereby ensuring sperm motility.

## Results

### CCDC28A is expressed in spermatids and is indispensable for male fertility

In light of the established role of CCDC28B in ciliogenesis^26,27^, we investigated whether CCDC28B or its paralogue, CCDC28A, might also play a role in sperm flagellum formation. To this end, we first examined the mRNA expression patterns in murine tissues using reverse transcription (RT)-PCR analysis. RT-PCR revealed similar tissue-specific expression of both proteins, with the highest expression levels observed in the brain and reproductive tissues (Fig. 1B). Single-cell RNA sequencing data from mouse testes indicated that *Ccdc28a* and *Ccdc28b* showed distinct, mutually exclusive expression patterns in the male reproductive system (Fig. 1C). Specifically, *Ccdc28b* was primarily expressed in somatic accessory cells, such as Leydig and Sertoli cells, whereas *Ccdc28a* was predominantly expressed in male germ cells, particularly in round and elongated spermatids (Fig. 1C).

The role of CCDC28A in male gametogenesis was further investigated by generating KO mice in which the coding exon 3 of *Ccdc28a* was deleted, leading to a frameshift mutation (*Ccdc28a*^*−/−*^) (Fig. S1A and S1B). To assess protein expression, rabbit antibodies against mouse CCDC28A were produced using polyhistidine (HIS)-tagged CCDC28A recombinant protein as the antigen (Fig. S1C). Western blot (WB) analysis of testis lysates using this anti-CCDC28A antibody verified the abolition of protein expression in the *Ccdc28a*^*−/−*^ testes (Fig. 1D). In mice, the first wave of spermatogenesis is completed within the first 35 days of postnatal development^29^. To determine the stage of spermatogenesis at which CCDC28A is expressed, we performed WB on testis extracts obtained from the testes at postnatal day (PD) 6 up to PD50. CCDC28A was weakly expressed in PD14 testes, with elevated expression observed from PD22 to PD50, which correlated with the development of round and elongated spermatids (Fig. 1E). This pattern was consistent with the mRNA expression profiles identified in the single-cell RNA sequencing data. (Fig. 1C). These data suggest that CCDC28A plays a role in spermatid development, particularly during spermiogenesis.

To assess fertility defects, *Ccdc28a*^*−/−*^ mice were continuously paired with wild-type (WT) mice for six months, commencing at two months of age. *Ccdc28a*^*−/−*^ females exhibited normal fertility, whereas *Ccdc28a*^*−/−*^ male mice were found to be completely infertile, failing to produce any litters when paired with WT females (Fig. 1F). Although the mRNA expression of *Ccdc28a* was detected in the ovary (Fig. 1B), multi-tissue WB analysis revealed that the protein expression was high in the testis and brain, but not in the ovaries (Fig. 1G). This finding underscores the importance of CCDC28A in male, but not female, fertility.

## CCDC28A prevents sperm tail bending and ensures motility

**Fig. 2:**
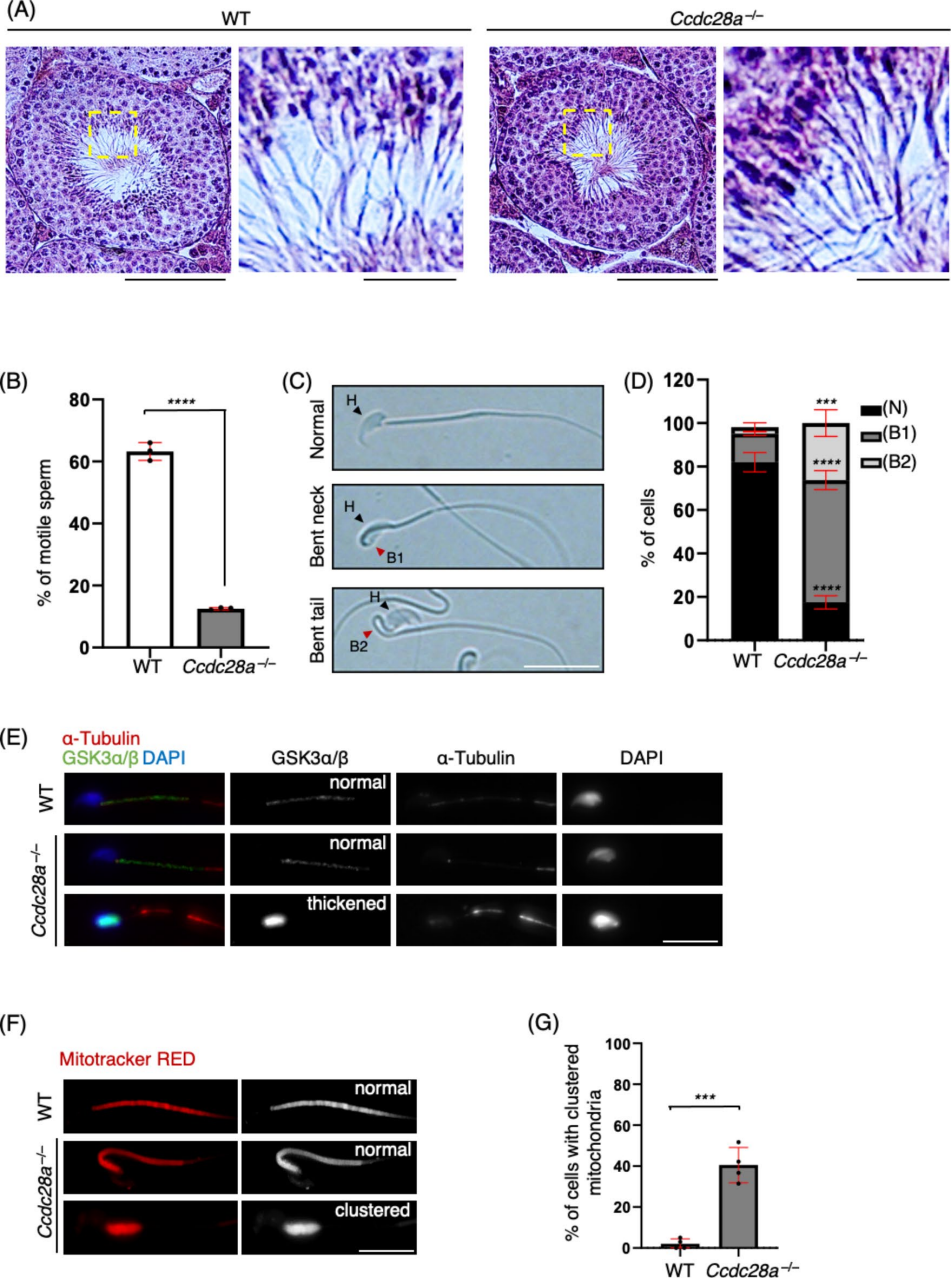
CCDC28A prevents sperm tail bending and ensures motility. (**A**) Testis sections from PD60 WT and *Ccdc28a*^*−/−*^ males stained with haematoxylin and eosin. The magnified images indicate spermatozoa. Scale bars: 50 μm (10 μm in the magnified panel). (**B**) The percentage of motile spermatozoa in cauda epididymis from PD60 WT and *Ccdc28a*^*−/−*^ mice. Spermatozoa were collected from three different mice for each genotype (n (WT) = 1899, n (*Ccdc28a*^*−/−*^) = 2235, n indicates the number of cells). Two-tailed *t*-test was used for statistical analysis. ****p < 0.0001. The mean values with SD are shown. (**C**) Cauda epididymal spermatozoa in bright field microscope from PD60 mice. Black arrowhead indicates sperm head (H), and red arrowhead indicates tail bending position (B1-bent neck, B2-bent midpiece). Scale bar: 5 μm. (**D**) The percentage of bent cauda epididymal spermatozoa in WT and *Ccdc28a*^*−/−*^ mice. N, normal; B1, bent neck; B2, bent midpiece. Spermatozoa were collected from five different mice for each genotype (n (WT) = 2037, n (*Ccdc28a*^*−/−*^) = 1312, n indicates the number of cells). Two-tailed *t*-test was used for statistical analysis. (****p < 0.0001, ***p < 0.001). The mean values with SD are shown. (**E)** Immunostaining of cauda epididymal spermatozoa from WT and *Ccdc28a*^*−/−*^ mice displaying normal midpiece (second panel) and bent-thickened midpiece (third panel). Scale bar: 5 μm. (**F**) Cauda epididymal spermatozoa from WT and *Ccdc28a*^*−/−*^ mice stained with MitoTracker Red CMXRos Dye, showing mitochondrial distribution pattern in tail midpiece. Second panel displays normal, and third panel displays clustered mitochondria. Scale bar: 5 μm. (**G**) Percentage of cauda epididymal spermatozoa from WT and *Ccdc28a*^*−/−*^ mice, displaying clustering of mitochondria in the midpiece. Spermatozoa were analysed for four different mice for each genotype (n (WT) = 137, n (*Ccdc28a*^*−/−*^) = 301, n indicates the number of cells). Two-tailed *t*-test was used for statistical analysis. ***p < 0.001. The mean values with SD are shown. Source numerical data are available in Source data.

Testicular samples from both WT and *Ccdc28a*^*−/−*^ mice were fixed in Bouin’s solution and subjected to haematoxylin and eosin staining for histological analysis. Examination of testis sections revealed the presence of spermatozoa in the *Ccdc28a*^*−/−*^ testis, indicating normal spermatogenesis (Fig. 2A). Further investigation of the cause of male infertility was conducted by isolating mature sperms from the cauda epididymis. Subsequent motility testing showed a significant reduction in sperm motility in *Ccdc28a*^*−/−*^ mice compared to that in WT controls, with only 12% of the sperms from *Ccdc28a*^*−/−*^ mice exhibiting tail movement, contrasting with the 63% motility observed in WT sperms (Fig. 2B).

Moreover, not only was motility reduced in *Ccdc28a*^*−/−*^ sperm, but also distinctive bending of the sperm tail was observed (Fig. 2C). The sperm tail was primarily bent at the neck (B1) or midpiece (B2) (Fig. 2C). Quantification of these bending phenotypes revealed a considerable increase in the mutant, with over 56% and 26% of KO sperms displaying bending in the neck region and midpiece, respectively (Fig. 2D). However, only 13% and 3% of WT sperms displayed bending in the neck region and midpiece, respectively. These bending phenotypes have been implicated in sperm motility defects^3,30^.

To further understand how sperm structure is affected, we examined the morphology of the sperm midpiece by immunostaining using the midpiece marker, glycogen synthase kinase 3α/β (GSK3α/β)^31^, as well as α-tubulin. In addition to the bending phenotype, we often observed abnormal shortening and thickening of the midpiece stained by GSK3α/β (Fig. 2E, second KO panel). The staining of F-actin, which is detected in the sperm head and along the tail, exhibited heightened intensity in the midpiece^32^, resembling that of GSK3α/β, further confirming the midpiece shortening and thickening in *Ccdc28a*^*−/−*^ sperms (Fig. S2). As the midpiece structure contains a mitochondrial sheath that surrounds the ODFs, we investigated whether this thickening phenotype was reflected in the mitochondrial distribution. The MitoTracker Red Dye staining revealed the presence of active mitochondria in KO sperms (Fig. 2F). Nevertheless, these mitochondria frequently appeared clustered, with quantification revealing that this phenotype is present in over 40% of *Ccdc28a*^*−/−*^ sperms (Fig. 2G). Taken together, it was concluded that *Ccdc28a*^*−/−*^ mice exhibit decreased sperm motility and consequent male infertility caused by aberrant sperm neck bending and midpiece defects.

## CCDC28A is indispensable for HTCA stability and the organization of mitochondrial sheath

**Fig. 3:**
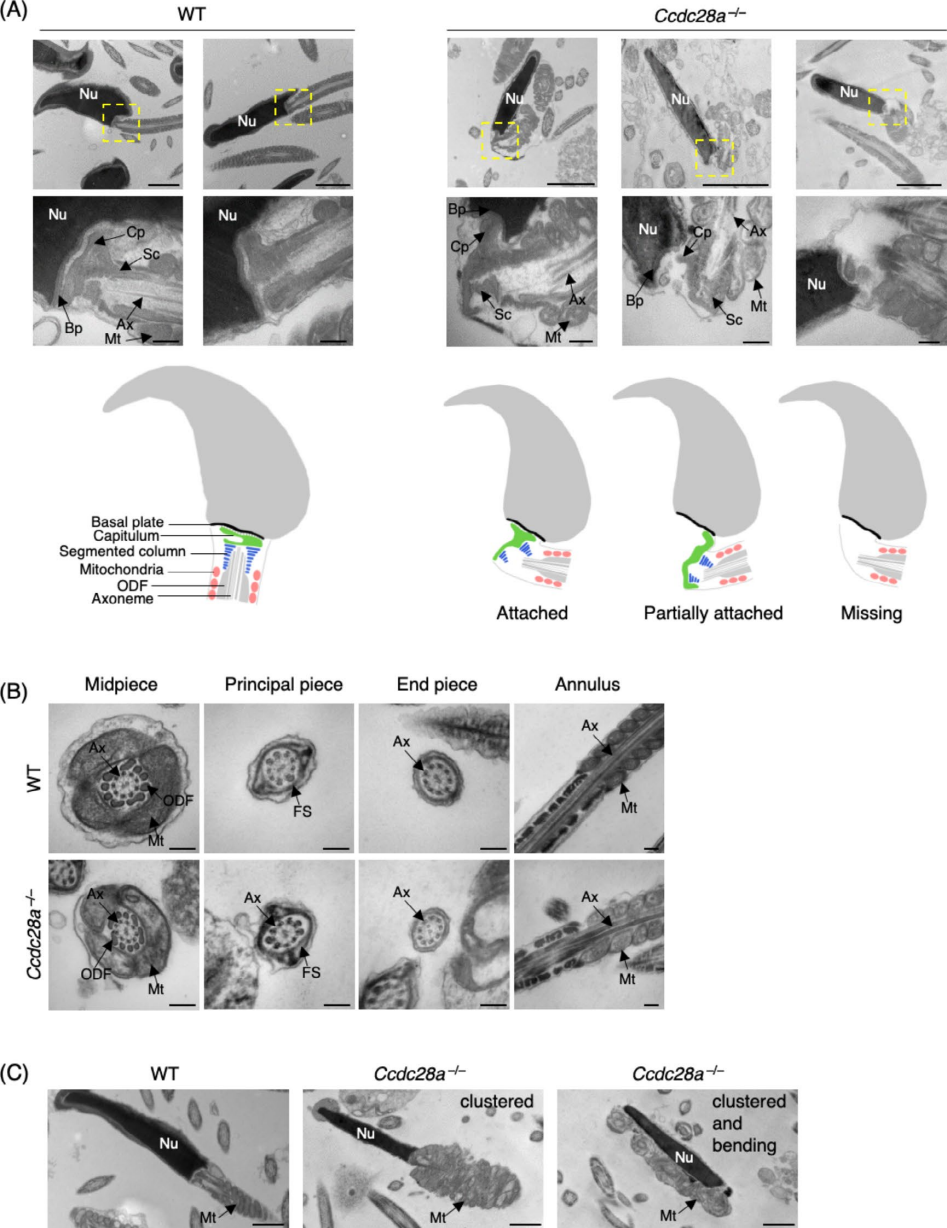
CCDC28A is indispensable for HTCA stability and the organization of mitochondrial sheath. (**A**) TEM images of WT and *Ccdc28a*^*−/−*^cauda epididymal spermatozoa. Nu, nucleus. Magnified images display the HTCA composing of Bp, basal plate; Cp, capitulum; Sc, segmented columns; Ax, axoneme; Mt, mitochondria. Scale bars: 2 μm (0.2 μm in the magnified panel). Schematics displaying HTCA organization in WT and *Ccdc28a*^*−/−*^mice spermatozoa showing the attached, partially attached and missing HTCA in KO mice. (**B**) TEM images of transversal section of the axoneme showing the regular microtubule organisation with nine microtubule doublets surrounding the central pair (9 + 2) in panels 1, 2 and 3 of WT and *Ccdc28a*^*−/−*^ cauda epididymal spermatozoa through mid to end piece. Ax, axoneme; ODF, outer dense fibers; Mt, mitochondria; FS, fibrous sheath. The fourth panel displays a longitudinal section of midpiece-principal piece junction of WT and *Ccdc28a*^*−/−*^ cauda epididymal spermatozoa. Scale bar: 0.2 μm. (**C**) TEM images of WT and *Ccdc28a*^*−/−*^ cauda epididymal spermatozoa displaying midpiece organisation. Nu, nucleus; Mt, mitochondria. Scale bar: 1 μm.

To investigate the underlying mechanisms of sperm tail bending in *Ccdc28a*^*−/−*^ mice, we utilised transmission electron microscopy (TEM) to assess the sperm structure (Fig. 3A). In WT samples, the sperm neck region consistently exhibited well-organised features, including a capitulum tightly attached to the basal plate, with well-defined segmented columns arranged beneath it. However, observations in the KO samples revealed a disrupted head-neck junction, characterised by imperfect attachment of the capitulum to the basal plate (Fig. 3A, first KO panel), often preceding neck bending. Furthermore, in some instances, complete detachment (Fig. 3A, second KO panel) or the absence (Fig. 3A, third KO panel) of the capitulum was observed in the KO samples. The missing attachment and structural defects of the capitulum were followed by disorganisation of all the structures associated with the neck, such as segmented columns, microtubules and mitochondria, localised close to the neck region.

Mutations in certain HTCA genes, such as *Cfap52*, not only result in the failure of connecting piece formation but also lead to remarkable defects in flagellar development, such as disordered axonemes and partial loss of ODFs^23^. Therefore, we next observed the flagellar structure through midpiece, principle and end piece to see if similar defects could be detected in *Ccdc28a*^*−/−*^ mice (Fig. 3B). Central to the flagellar structure is the axoneme, which consists of nine outer doublet microtubules and a central microtubule pair (Fig. 3B)^3^. In the midpiece, the axoneme is encased by a mitochondrial sheath and ODFs^33^, whereas in the principal piece, it is surrounded by a fibrous sheath and ODFs (Fig. 3B)^34^. No morphological disparities were detected in the axoneme and ODF organisation in the midpiece, principal piece and endpiece of KO spermatozoa (Fig. 3B). These data suggest that the flagellar structure remains unaffected in the *Ccdc28a*^*−/−*^ mice. Consistent with the MitoTracker Red Dye staining data, *Ccdc28a*^*−/−*^ spermatozoa frequently showed abnormal clustering of mitochondria at the neck region (Fig. 3C, first KO panel), which sometimes accompanied the tail bending phenotypes (Fig. 3C, second KO panel). Thus, we concluded that the structural anomaly observed in the absence of CCDC28A appears to be confined to the HTCA and mitochondrial sheath, whereas the flagellar structure remains unaffected.

## CCDC28A localizes to both the manchette and the HTCA

To investigate the localisation of CCDC28A, we performed immunostaining for endogenous CCDC28A using polyclonal antibodies. Focal signals were observed in the testicular spermatids, and signals were also detected at the midpiece of the epididymal spermatozoa (Fig. S3A and S3B). However, the signals observed in the WT samples were not completely abolished in the KO samples, likely owing to overlapping nonspecific signals, thereby precluding the use of this antibody for immunofluorescence analysis. (Fig. S3A and S3B).

To further explore CCDC28A localisation, we examined exogenous protein expression in the testes using an in vivo electroporation technique^35^. We electroporated pCAG-*gfp*-*Ccdc28a* plasmid DNA into live mouse testes on PD24. After 13 days, the mice were sacrificed, and CCDC28A localisation was examined in the testis cell spreads.

**Fig. 4:**
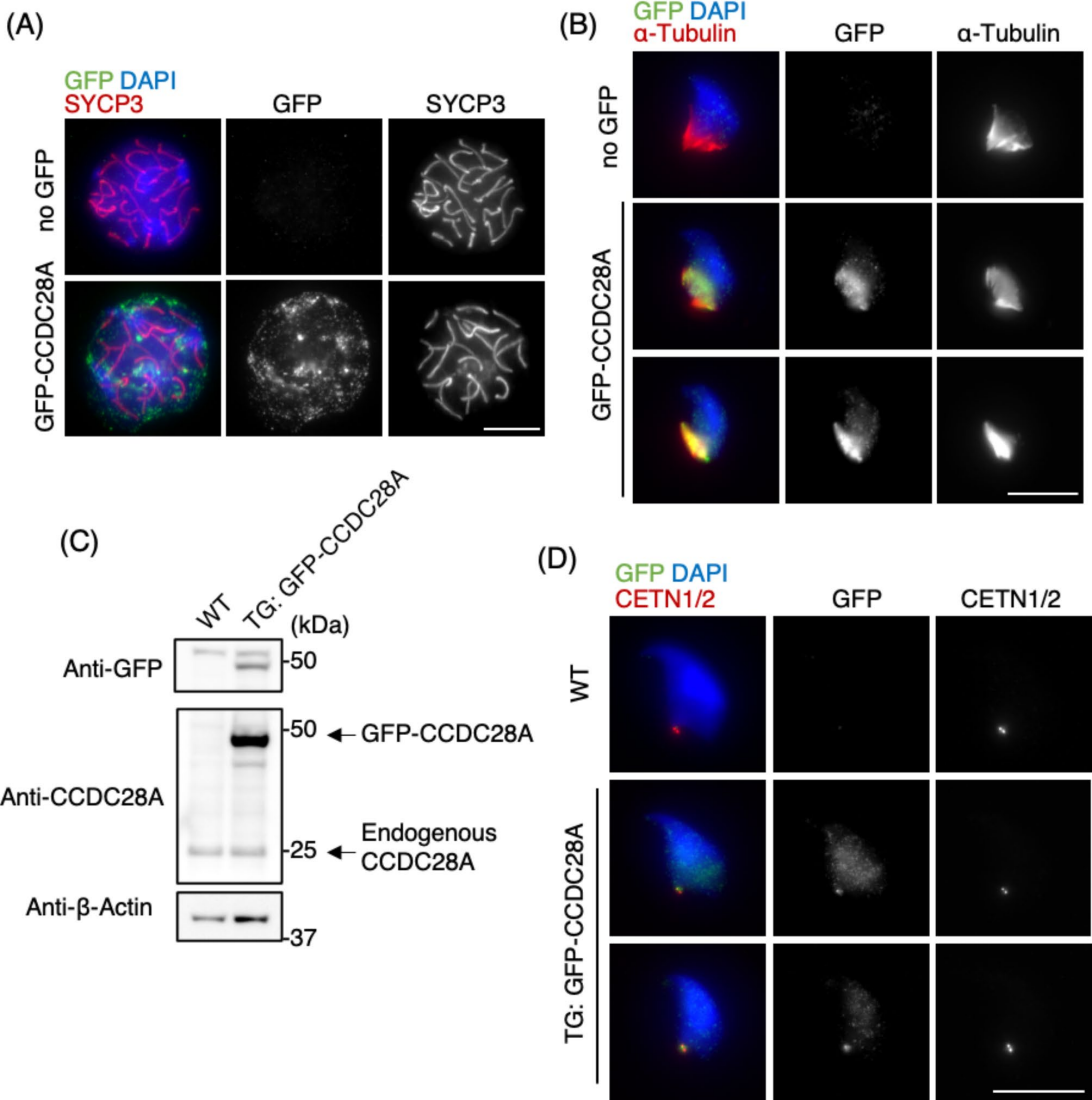
CCDC28A localizes to both the manchette and the HTCA. (**A**) Immunostaining of WT spermatocytes in early-pachytene expressing GFP-CCDC28A by in vivo electroporation, stained with the indicated antibodies. Scale bar: 5 μm. (**B**) Immunostaining of WT elongating spermatids expressing GFP-CCDC28A by in vivo electroporation, stained with the indicated antibodies. Scale bar: 5 μm. (**C**) Immunoblot of testis extracts of WT and *gfp*-*Ccdc28a* transgenic mice, blotted with the indicated antibodies. The single membrane was stripped and sequentially blotted with the indicated antibodies. (**D**) Immunostaining of elongating spermatids from WT and *gfp*-*Ccdc28a* transgenic mice, stained with the indicated antibodies. The middle and lower rows show early and late elongation stages, respectively. Scale bar: 5 μm. Unprocessed blots are available in Source data.

Exogenously expressed green fluorescent protein (GFP) tagged-CCDC28A was detectable in spermatocytes but exhibited amorphous cytoplasmic-like signals and did not display specific localisation patterns (Fig. 4A). However, in elongating spermatids, exogenous GFP-CCDC28A was found specifically localised to the manchette (Fig. 4B). The manchette mechanically shapes the distal half of the sperm head by constricting the nucleus while progressively ratcheting it. Additionally, the manchette is essential for HTCA formation, delivering proteins to the HTCA via intramanchette transport^6,16^. Consequently, HTCA abnormalities have been observed in mice lacking manchette-localising proteins, such as IFT88^36^, CCDC42^24,37^, FAM46C^38^and ODF1^8,39^.

To examine the localisation of CCDC28A in a developmental stage-specific manner, we generated transgenic mice stably expressing GFP-CCDC28A. WB analysis of testicular extracts confirmed the expression of GFP-CCDC28A in transgenic mice, and its expression level was considerably higher than that of the endogenous protein (Fig. 4C). Subsequent examination of GFP-CCDC28A localisation in elongating spermatids revealed a nuclear signal, predominantly during the initial stages between steps 10 and 13 of elongation, with a pronounced signal observed in the manchette (Fig. 4D, middle row). In the later stages of elongation after step 13, which were characterised by hook-shaped and condensed DAPI-stained nuclei, the signal was concentrated near the HTCA, adjacent to the centriolar marker CETN1/2 (Fig. 4D, lower row). These observations indicate a dynamic localisation pattern of CCDC28A throughout spermatid elongation, which aligns with the potential role of CCDC28A in HTCA formation and stability.

## Discussion

The distinct expression patterns of *Ccdc28a* and its paralogue *Ccdc28b* suggest diverse roles within the male reproductive system. Although *Ccdc28b* is primarily expressed in somatic accessory cells, *Ccdc28a* is expressed in germ cells, specifically in spermatids. Although *Ccdc28b *KO mice have been reported to be fertile^28^, male mice lacking *Ccdc28a* are completely infertile. Histological analysis of testis sections from *Ccdc28a* KO mice did not reveal any noticeable defects in spermatogenesis progression. Motility testing of mature sperms isolated from the epididymis revealed a significant reduction in sperm motility in *Ccdc28a* KO mice, which was accompanied by distinctive bending and twisting of the sperm tail, primarily in the neck region or midpiece. This motility reduction and neck bending are linked to disruptions in the head-neck junction, as TEM demonstrates partial or complete detachment of the capitulum from the basal plate, leading to the disorganisation of structures associated with the neck. These findings indicate that CCDC28A is crucial for maintaining HTCA stability.

A comparable phenotype of sperm neck bending, in which the sperm heads are fully bent backward to the extent that the head tip points toward the tail tip, has been documented in other KO models such as *Spem1 *KO^40^. In *Spem1* KO mice, neck bending was attributed to retained cytoplasmic components that mechanically obstructed the straightening of the sperm head and elongation of the growing tail. This obstruction resulted in the bending of the head within the neck, followed by wrapping of the head with the neck or the midpiece of the sperm tail. TEM analysis revealed numerous small interconnected membranous vacuoles, indicative of cytoplasmic retention between the head and tail structures in spermatozoa from *Spem1* KO mice. In *Ccdc28a* KO mice, we did not observe such membranous structures. This suggests that the bending of the sperm neck observed in *Ccdc28a* KO mice may arise solely as a consequence of structural detachment within the HTCA, rather than cytoplasmic retention.

Sperm mitochondria are essential for functional spermatozoa because they supply energy to support their motility^41^. *Ccdc28a* KO mice exhibited a thickened midpiece structure containing clustered functional mitochondria, as revealed by the MitoTracker Red Dye staining. Their abnormal localisations in the *Ccdc28a*^*−/−*^mice can structurally impair spermatozoa. Patients with asthenozoospermia display similarly thickened sperm midpieces^42^. Despite maintaining full oxidoreductive capability, sperm from these patients demonstrated non-progressive movement or immotility, suggesting that asthenozoospermia may not arise solely from energetic disruptions within sperm mitochondria^42^. Rather, diminished sperm motility may be associated with deformities in the mitochondrial sheath housing functional mitochondria, which structurally support the midpiece during tail movement^43^. Bending of the sperm neck affects the distribution of mitochondria near the sperm neck, potentially contributing to sperm motility defects, in addition to disruptions in the head-tail connection.

Localisation studies elucidated the dynamic distribution of CCDC28A during spermatid elongation. The expression of exogenous CCDC28A in elongating spermatids revealed its specific localisation to the HTCA and manchette, which is critical for the distribution of proteins essential for HTCA formation^6,16,43^. It is interesting to note that in preliminary data from a yeast two-hybrid screen using mouse *Ccdc28a* (amino acids 1-184) as bait against a mouse testis library, we found *Bag6* (BCL2-associated athanogene 6) as one of the most frequently identified genes (Fig. S4A). *Bag6* has been previously implicated in spermatogenesis and male fertility, with *Bag6 *exon 24 KO resulting in impaired ultrastructure and morphology of sperm^22^. TEM analysis of *Bag6 *exon 24 KO revealed disrupted connections between the sperm head and neck, as well as substantial loss of ODFs in the principal piece of sperm flagella^22^. A potential interaction between CCDC28A and BAG6 may be important for regulating the structural integrity of HTCA, warranting further experimental validation to elucidate its functional importance.

Overall, the findings of this study underscore the importance of CCDC28A in male fertility, particularly in sperm motility. This conclusion is consistent with a recent report on *Ccdc28a *KO mice that were independently generated using CRISPR-Cas9-mediated gene editing^44^. The involvement of this protein in sperm tail formation and its dynamic localisation during spermatid elongation highlight its importance in the intricate process of spermatogenesis. Future studies exploring the molecular mechanisms underlying CCDC28A function and its interactions with other proteins involved in sperm tail formation and motility will provide further insights into male infertility and potential therapeutic targets for reproductive disorders.

## Methods

### Mice

*Ccdc28a* KO mice were generated from the embryonic stem cell line C57BL/6N-*Ccdc28atm3b(KOMP)*^*Wtsi/JMmucd*^. The *Ccdc28a* allele was genotyped using the following primers: WT-forward; 5′- GCTCGACTTCGAAAACTACATGAGG − 3′, WT-reverse; 5′- CACTTCACAGTAAAATGCAGCAAGC − 3′, KO-forward; 5′- GCTACCATTACCAGTTGGTCTGGTGTC − 3′ KO-reverse; 5′- TGTTTTTGAGGCAGGTTTCAGGTAGC − 3′ (Fig. S1B). All WT and KO mice were congenic with a C57BL/6 N background. The animal experiments were approved by the Regional Ethics Committee of Gothenburg, which is governed by the Swedish Board of Agriculture (#1316/18 and #4851/23), and carried out in accordance with the institutional guiding principles for the care and use of laboratory animals. The study was carried out in compliance with the ARRIVE guidelines (https://arriveguidelines.org). *Gfp-Ccdc28a* transgenic mice were generated at the Karolinska Center for Transgene Technologies (Stockholm, UK). The 3.5-kb DNA fragment cleaved from the pCAG-*gfp*-*Ccdc28a* vector using *Spe* I and *Hind* III was purified and used to generate transgenic mice. Mice were euthanized by cervical cord dislocation.

## Antibodies

The following antibodies were used: rabbit antibodies against CCDC28A (generated in this study) IF and WB 1:1000, GFP (Invitrogen, A11122, 2339829) IF and WB 1:1000, GSK-3α/β (Cell Signalling Technology; 5676T, 7) IF 1:1000; Mouse antibodies against α-tubulin (Abcam, ab7291, GR3398636-5) IF and WB 1:2000, β-actin (Sigma-Aldrich; A2228, 118M4829V) 1:2000 (WB); a rat antibody against CETN1/2 (BioLegend; 698602, B333787) IF 1:200; a chicken antibody against SYCP3^45^ IF and WB 1:3000.

### Antibody production

The cDNA encoding full-length *Ccdc28a* was cloned into the pET28c + vector (Millipore). The HIS-tagged recombinant protein was expressed in *E. coli* BL21 (DE3) cells, solubilised in a buffer containing 600 mM NaCl, 30 mM imidazole, 20 mM Tris-HCl (pH 7.5) and 0.1% Triton X-100 and purified using Ni-NTA resin (Qiagen). Resins were washed with 600 mM NaCl, 30 mM imidazole, 20 mM Tris-HCl (pH 7.5) and 0.1% Triton X-100, and the proteins were eluted using 600 mM NaCl, 500 mM imidazole and 20 mM Tris-HCl (pH 7.5) buffer. Recombinant proteins were dialysed in phosphate-buffered saline (PBS) and used to immunise the animals. The polyclonal antibody against CCDC28A was affinity purified using antigen-coupled Sepharose beads (GE Healthcare).

### Reverse transcription PCR

Total RNA was isolated from the tissues using an RNeasy Mini Kit (Qiagen). Reverse transcription using 0.09 µg total RNA was performed, and cDNAs were generated by iScript reverse transcription super mix (Bio-Rad). Products were amplified by denaturation at 95 °C for 30 s, 35 cycles of 95 °C for 10 s, 60 °C for 15 s and 72 °C for 30 s using the appropriate primers and standard Taq DNA polymerase (New England Biolabs). The primers used were: *Ccdc28a*-forward 5′- CTGCAGGCGTTTGGAAATGA − 3′, *Ccdc28a*-reverse 5′- CGTCTGCCAAGTGCAGTTTC − 3′, *Ccdc28b*-forward 5′- AGAAACGGAGTCCTAAGCCC-3′, *Ccdc28b*-reverse 5′-CTCCTTGCCTGCTCTCTTGAA-3′, *Gapdh*-forward 5′- TTCACCACCATGGAGAAGGC-3′, *Gapdh* -reverse 5′- GGCATGGACTGTGTGGTCATGA − 3′.

### Single-cell RNA sequencing transcriptome analysis

Single-cell RNA-sequencing data for young mouse testes were obtained from previously published reports (E-code E-MTAB-6946)^46^. Sequence reads from PD5–25 WT mouse testes were aligned to the reference mouse genome data (mm10) using the 10x Genomics Cell Ranger count pipeline version 6.0.2 with default settings. The multiplexed samples were aggregated using the Cell Ranger aggr pipeline with default settings. Cell populations were clustered into seven groups using the k-means clustering method and plotted using uniform manifold approximation and projection (UMAP) with 10x Genomics Loupe Browser software version 6.2. The cell types or spermatogenic developmental stages of clustered populations were identified based on the expression patterns of known stage-specific marker genes, including *Stra8* for preleptotene spermatocytes; *Sycp3*, *Piwil1*, *Spo11* and *Dmc1* for meiotic spermatocytes; *Rec8* for spermatocytes and round spermatids; *Sox9* and *Wt1* for Sertoli cells and *Cyp11a1*, *Hsd3b1* and *Insl3*for Leydig cells^47^.

### Histological analysis

Testes were fixed in Bouin’s fixative for 24 h at 20–25 °C, serially dehydrated and embedded in paraffin blocks. Slices of 8 μm thickness were stained with haematoxylin and eosin.

### Sperm motility assay

The cauda epididymis was dissected from adult mice. Sperms were squeezed out from the cauda epididymis into pre-warmed Human Tubal Fluid Medium (HTF) (Sigma-Aldrich; MR-070-D) at 37 °C. The incubated sperm medium was then diluted to the final ratio of 1:500, and 10 µl were placed onto a Neubauer Improved Haemocytometer Counting chamber (Hecht Assistant) for counting.

### MitoTracker Red Dye staining

The cauda epididymis was dissected from adult mice. Sperms were squeezed out from the cauda epididymis into prewarmed HTF (Sigma-Aldrich; MR-070-D). MitoTracker Red CMXRos (Invitrogen, M7512) was added at a working concentration of 200 nM and incubated for 10 min at 37 °C in the dark. Five microlitres were placed onto a glass slide covered with a coverslip and incubated at 4 °C for 5 min for the sperm to stop moving. Slides were observed and imaged using a fluorescence microscope.

### Immunostaining of cauda epididymal sperms

The cauda epididymis was dissected from adult mice. Sperms were squeezed out of the cauda epididymis into PBS. The cells were placed on a glass slide and dried at 37 °C for 20 min. Cells were fixed with 4% paraformaldehyde in PBS for 10 min and washed several times with PBS. For immunostaining, the slides were incubated with primary antibodies in PBS containing 5% bovine serum albumin (BSA) overnight by incubation with the following secondary antibodies for 1 h at 20–25 °C: donkey anti-rabbit alexa 488 (1:500; A21206, 2376850; Invitrogen) and donkey anti-mouse alexa 594 (1:500; A21203, 2352146; Invitrogen). The slides were washed with PBS and mounted in Vectashield medium with DAPI (Vector Laboratories).

### Immunostaining of spermatocytes

The testes were minced with flathead forceps in PBS, washed several times in PBS and resuspended in a hypotonic buffer (30 mM Tris (pH 7.5), 17 mM trisodium citrate, 5 mM EDTA, 2.5 mM dithiothreitol (DTT), 0.5 mM phenylmethylsulfonyl fluoride (PMSF) and 50 mM sucrose). After 10 min, the sample was centrifuged, and the supernatant was aspirated. The pellet was resuspended in 200 mM sucrose. After 10 min, equal volumes of fixation buffer (1% paraformaldehyde and 0.15% Triton X-100) were added. The cells were then placed on a glass slide, fixed for 2 h at 20–25 °C and air-dried. For immunostaining, the slides were incubated overnight with primary antibodies in PBS containing 5% BSA, followed by incubation with the following secondary antibodies for 1 h at 20–25 °C: donkey anti-rabbit alexa 488 (1:500; A21206, 2376850; Invitrogen), donkey anti-mouse alexa 594 (1:500; A21203, 2352146; Invitrogen), goat anti-chicken alexa 594 (1:500; Invitrogen; A78951) and donkey anti-rat alexa 594 (1:500; A21209, 1807726). The slides were washed with PBS and mounted in Vectashield medium with DAPI (Vector Laboratories).

### Microscopy

Images were obtained using a microscope (Olympus IL-X71 Delta Vision; Applied Precision) equipped with 100× NA 1.40 and 60× NA 1.42 objectives, a camera (CoolSNAP HQ; Photometrics) and softWoRx 7.2.1 acquisition software (Delta Vision). All the acquired images were processed using Photoshop 25.6.0 (Adobe).

### Transmission electron microscopy (TEM) of epididymal sperms

Sperms were collected from the cauda epididymis and dispersed in Hanks’ balanced salt solution (Sigma-Aldrich, H8264). The samples were fixed in 100 mM cacodylate buffer containing 2.5% glutaraldehyde and 4% paraformaldehyde (pH 7.2) for 2 h at 20–25 °C. The fixed sperms were then pelleted at 1000 rpm, rinsed twice with the same cacodylate buffer and stored in the buffer until further processing. The samples were washed thrice with cacodylate buffer and pelleted after each wash. Post-fixation was performed using 1% osmium tetroxide for 1 h at 20–25 °C. Subsequently, the samples were washed four times with cacodylate buffer at 4 °C. Pre-embedding staining was performed using 1% uranyl acetate for 1 h at 4 °C, washed twice with distilled water and dehydrated using a graded ethanol solution. The pelleted sperm samples were incubated twice with propylene oxide for 10 min at 20–25 °C. The samples were embedded in Embed 812 resin (EMS #14120) and polymerised for 48 h at 60 °C. Ultrathin sections, approximately 60-nm thick, were collected on Formvard resin (Ted Pella)-coated copper grids (Gilder Grids Standard Square Mesh, EMS). Sections were contrasted for 20 min with uranyl acetate for 10 min and lead citrate before examination under a transmission electron microscope at 100 kV (Jeol 1010). This detailed preparation protocol ensured high-quality visualisation of the epididymal sperm structures, allowing for precise ultrastructural analysis using TEM.

### Exogenous protein expression in the testis

Plasmid DNA was electroporated into live mouse testes using in vivo electroporation technique^35^. Briefly, male mice at PD26 were anaesthetised with ketamine-xylazine, and the testes were pulled from the abdominal cavity. Plasmid DNA (10 µl of 5 µg/µl solution) was injected into each testis using glass capillaries under a stereomicroscope (M165C; Leica). Testes were held between a pair of tweezer-type electrodes (CUY21; BEX), and electric pulses were applied four times and again four times in the reverse direction at 35 V for 50 ms for each pulse. The testes were then returned to the abdominal cavity, and the abdominal wall and skin were closed with sutures. The testes were removed 24 h or 13 days after electroporation, and immunostaining was performed.

### Yeast two-hybrid screening

Yeast two-hybrid screening was performed using Hybrigenics Services (Paris, France). The coding sequence of Mus musculus *Ccdc28a* (aa 1-184) was amplified by PCR and cloned into pB27 as a C-terminal fusion with the LexA DNA-binding domain (*N-LexA-Ccdc28a-C*). The construct was confirmed by sequencing and used as bait to screen a mouse testis cDNA library (Mouse Testis_RP1). In total, 115 million clones were identified.

#### **Quantification and statistical analysis**

Statistical analyses were conducted using GraphPad Prism (Version 10.1.0) and Microsoft Excel (16.89.1). No formal statistical methods were applied to predetermine the sample sizes, which were selected based on conventions in the field and are consistent with sample sizes used in comparable studies. For comparisons of two groups, two-tailed Student’s t test was used (Figs. 1F and 2B, D and G). Statistical details for each experiment are provided in the corresponding figure legends and figures.

## Electronic supplementary material

Below is the link to the electronic supplementary material.

Supplementary Material 1

Supplementary Material 2

## Data Availability

All data are available in the main text or supplementary materials. The source data are provided in this study. All other data supporting the findings of this study are available from the corresponding author upon request.
