# Supplementary material for: CCDC28A deficiency causes head-tail coupling defects and immotility in murine spermatozoa
Source: Sci Rep. 2024 Nov 5;14:26808. doi: 10.1038/s41598-024-78453-9 (PMC11538371; doi:10.1038/s41598-024-78453-9)
Supplement: Supplementary file 2 — Supplementary Material 2 [file 41598_2024_78453_MOESM2_ESM.pdf]

Supplementary Information for

CCDC28A Deficiency Causes Head-Tail Coupling Defects and Immotility in Murine Spermatozoa

Nena Stojanovic, Rosario Ortiz Hernández, Nayeli Torres Ramírez, Olga Margarita Echeverría Martínez, Abrahan Hernández Hernández and Hiroki Shibuya

The PDF file includes:

Figs. S1 to S4

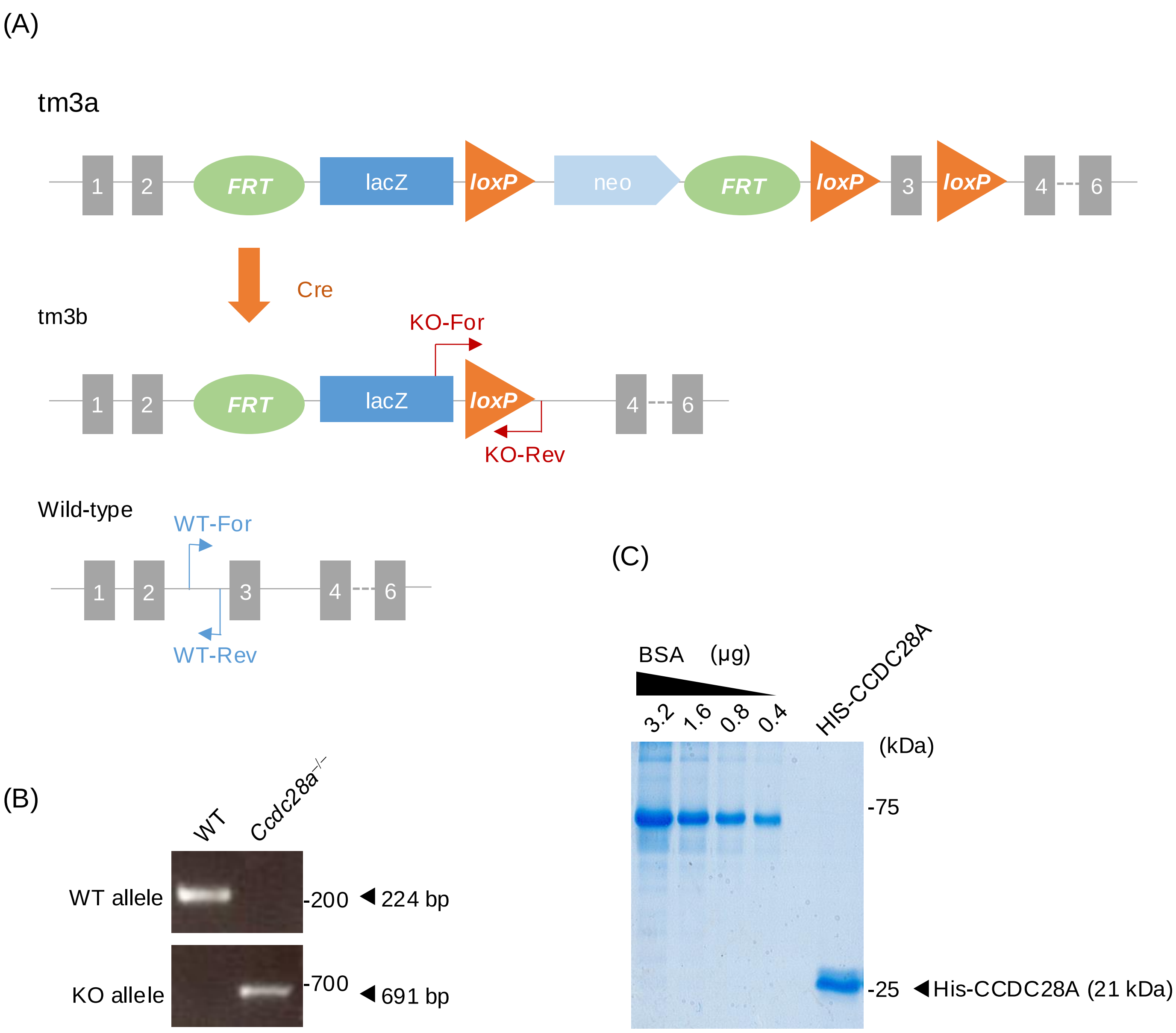

Figure S1. ***Ccdc28a*** $^{-/-}$ **allele and antigen purification**

(**A**) The *Ccdc28a* gene trap allele. Rectangles represent exons. Flippase recognition target; FRT. Neomycin resistance cassette; Neo. Splicing accepter; SA and Polyadenylation signal; pA. (**B**) Genotyping result of WT and *Ccdc28a* $^{-/-}$ mice, using WT and KO primers. The position of primers is shown in (**A**). (**C**) SDS-PAGE analysis of affinity purified His-tagged CCDC28A protein from *E. coli* cell lysate. Bovine serum albumin (BSA) gradient was used to determine concentration of the purified antigen. Unprocessed gel images are available in Source data.

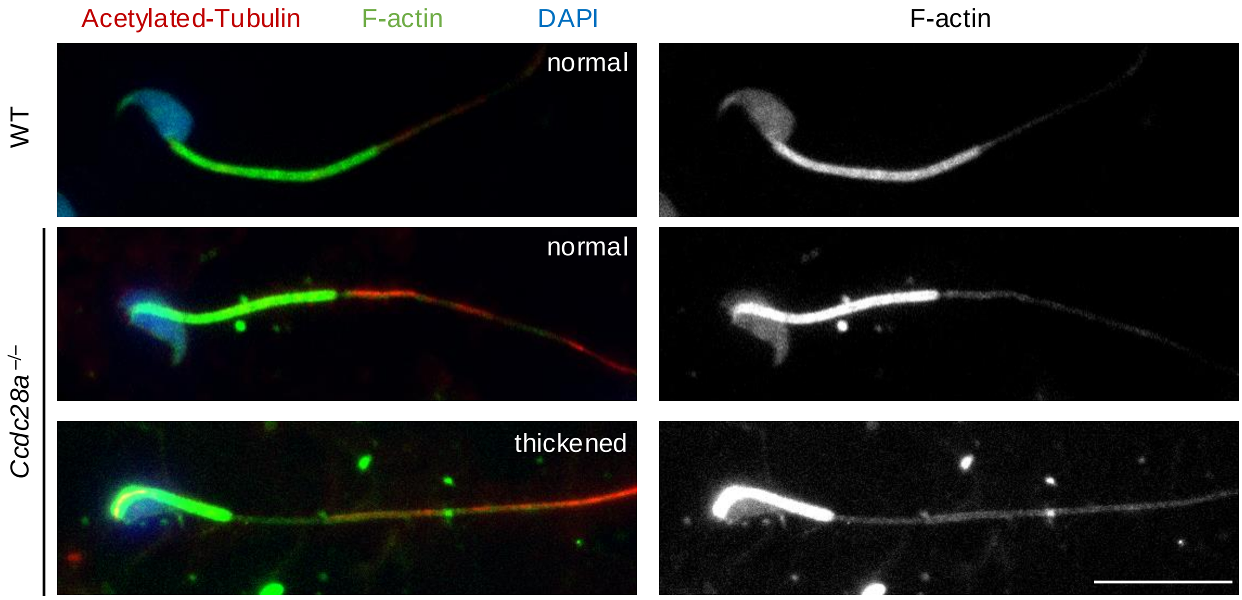

Figure S2. CCDC28A is required for proper midpiece organization

Phalloidin staining of cauda epididymal spermatozoa from WT and *Ccdc28a*$^{-/-}$mice displaying bent non-thickened midpiece (second panel) and bent-thickened midpiece (third panel) spermatozoa. Scale bar: 5 μm.

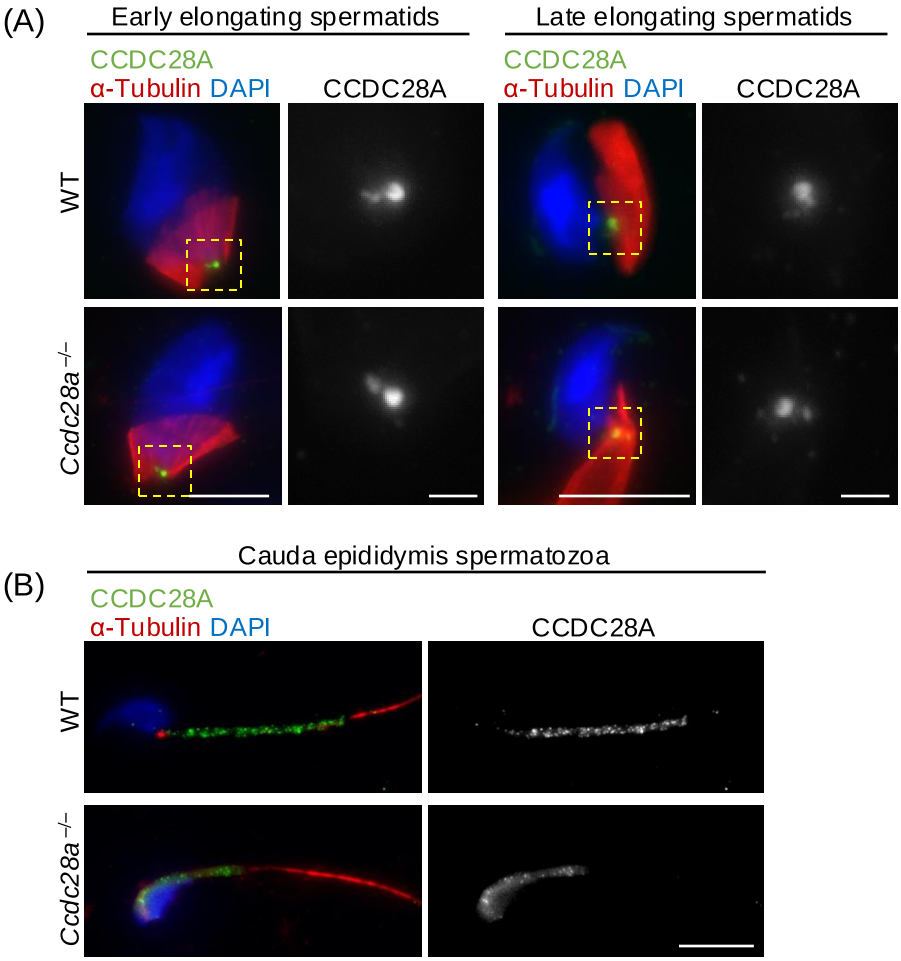

Figure S3. Immunostaining of endogenous CCDC28A

(**A**) Immunostaining of early and late elongating spermatids from WT and *Ccdc28a*$^{-/-}$ testes with the CCDC28A polyclonal antibody. Scale bar: 5 µm (1 µm in the magnified panel).

(**B**) Immunostaining of cauda epididymal spermatozoa from WT and *Ccdc28a*$^{-/-}$ mice, with generated CCDC28A antibody. Scale bar: 5 µm.

Name	NCBI ref #	Protein full name	# Hits
BAG6	NP_001391256.1	BCL2-associated athanogene 6	13
VCL	NP_033528.3	Vinculin	10
ANAPC4	NP_077175.1	Anaphase-promoting complex subunit 4	4
COPS6	NP_036132.1	COP9 signalosome complex subunit 6	4
USP12	NP_035799.1	Ubiquitin carboxyl-terminal hydrolase 12	3
TRIM13	NP_001157692.1	Tripartite motif containing 13	2

Figure S4. Yeast-two-hybrid screening results

Mouse testis cDNA library was used as prey, with *Ccdc28a* (aa 1-184) used as bait. Proteins which displayed good to very high confidence in interaction (assessed by the Hybrigenics Services, S.A.S., Paris, France) are presented.
